# Factors associated with applying to graduate/professional degrees for students engaged in undergraduate research experiences at minority serving institutions

**DOI:** 10.3389/feduc.2025.1589105

**Published:** 2025-06-08

**Authors:** Lourdes E. Echegoyen, Kala M. Mehta, Karsten Hueffer, Gabriela Chavira, Jacob D. Kagey, Thomas E. Keller, Kathleen M. Morgan, Stephen B. Aley, Chi-Ah Chun, Payam Sheikhattari, Amy Wagler

**Affiliations:** 1 Department of Chemistry & Biochemistry, Campus Oce of Undergraduate Research Initiatives, The University of Texas at El Paso, El Paso, TX, United States; 2 Department of Epidemiology and Biostatistics, University of California San Francisco, San Francisco, CA, United States; 3 College of Natural Science and Mathematics, The University of Alaska Fairbanks, Fairbanks, AK, United States; 4 California State University, Northridge, CA, United States; 5 Department of Biology, University of Detroit Mercy, Detroit, MI, United States; 6 School of Social Work, Portland State University, Portland, OR, United States; 7 Department of Chemistry, Xavier University of Louisiana, New Orleans, LA, United States; 8 Department of Biological Sciences, The University of Texas at El Paso, El Paso, TX, United States; 9 Department of Psychology, California State University-Long Beach, Long Beach, CA, United States; 10 School of Community Health and Policy, Morgan State University, Baltimore, MD, United States; 11 Department of Public Health Sciences, The University of Texas at El Paso, El Paso, TX, United States

**Keywords:** undergraduate students, minority students, undergraduate research, applying to graduate/professional school, biomedical

## Abstract

**Introduction:**

This study investigates the impact of undergraduate research experiences on applications to graduate and professional programs, particularly for underrepresented minority students at Minority Serving Institutions (MSIs).

**Methods:**

The study analyzes data collected at 10 MSIs participating in the NIH BUILD program to understand the relationship between research participation (in formal programs vs. informal research), student demographics, science self-efficacy, GPA, and application to advanced degree programs.

**Results:**

Results indicate that undergraduate research participation, especially in formal programs for extended periods of time, positively influences applications to graduate/professional programs, with similar outcomes observed across underrepresented minority and non-minority students.

**Discussion:**

Findings indicate that organized programs in biomedical research training significantly increase the probability of students applying to graduate or professional programs when programs span more than 12 months. This has implications for the design and implementation of biomedical research training programs, especially at MSIs.

## 1 Introduction

It has been a long-standing goal of the scientific community to recruit, train and retain a diverse scientific workforce (Freeman and Huang, 2014; Page, 2019; Swartz et al., 2019). The different perspectives brought by team members from different backgrounds and experiences provide opportunities to develop the innovative thinking needed to solve the most complex problems plaguing today’s society (Freeman and Huang, 2014; Page, 2019; Swartz et al., 2019). In addition, science, technology, engineering and mathematics (STEM) jobs are better compensated than many other occupations, which for individuals from low socioeconomic backgrounds and minoritized groups can bring about economic stability, generational wealth and social mobility (Benish, 2018; Falkenheim and Alexander, 2023). Benish (2018) and Deitz and Freyman (2024) have pointed to the importance of recruiting, training, and retaining a diverse scientific workforce, which has become a long-standing goal of the scientific community (Freeman and Huang, 2014; Page, 2019; Swartz et al., 2019). Yet entry into the scientific workforce has been challenging to achieve, especially for first-generation college students, (Bettencourt et al., 2020; Fabiano, 2022) and students from systemically minoritized communities, who are less likely to persist in STEM postsecondary majors (Fry et al., 2021; McFarland et al., 2017). Consequently, attaining long-term parity between the demographics of the US population and the demographics of the scientific workforce has been challenging, especially considering that first-generation college students and students from systemically minoritized communities are less likely to persist in science, technology, engineering, and mathematics (STEM) postsecondary majors (Bettencourt et al., 2020; Fabiano, 2022; Fry et al., 2021; McFarland et al., 2017).

To address these disparities, institutions of higher education have developed initiatives to increase access to STEM education, such as outreach and engagement of high school students and teachers, assistance with curricular modifications, developmental programs to improve preparedness, and scholarship and loan programs that provide financial assistance to socioeconomically underprivileged students (Espinosa, 2011; Estrada et al., 2016; Means et al., 2016). All of these have allowed for an increased enrollment of minoritized students in higher education by 20% over the past 25 years (Musu-Gillette et al., 2017). Despite increases in overall STEM degree enrollments from associate’s to doctoral degrees over the past decade (Falkenheim and Alexander, 2023), the degree completion rates remain low for many historically underrepresented groups in STEM compared to the general U.S. population ages 20–34 years old. For example, Black, Latinx and Native American/Alaska Native students graduate from college at lower rates than White students (Riegle-Crumb et al., 2019). Black, Latinx and Native American/Alaska Native individuals make up only 9.2, 18.3 and 0.4% of all STEM bachelor’s degrees awarded and 8.2, 14.8 and 0.3% of the STEM workforce, respectively, even though they make up 14.3, 21.7 and 0.8% of people ages 20–34 in the United States (Deitz and Freyman, 2024). As the U.S. becomes increasingly diverse, it is imperative to find ways to increase retention and graduation rates among minoritized students in STEM.

A closer examination of what happens to students majoring in STEM has revealed that minoritized students are leaving the STEM majors before they graduate, a disparity also known as the “push-out” (Vargas et al., 2021). Riegle-Crumb et al. (2019) found that while there is no significant difference in students majoring in STEM in their first year of college, with 18% of Black students, 20% of Latinx students, and 19% White students declaring a STEM major, about 37% of the Latinx students and 40% of the black students switched majors compared to 29% of the White students before they graduate. Another recent study on first-year STEM inclusion found that institutional and environmental factors contribute to students being pushed out of STEM majors (Wagler et al., 2024). They also found that more minoritized students in STEM majors left their undergraduate institutions without earning any college degrees (26% Black, 20% Latinx, 13% White). Factors contributing to the push-out of minoritized students in STEM include limited academic preparation, poor performance in introductory STEM courses (Hatfield et al., 2022), fewer same-race peers and less family member support (Strayhorn et al., 2013), less faculty contact and mentorship (Aruguete, 2017; Chang et al., 2014), less emotional support and encouragement from family members (Chang et al., 2014; Cole and Espinoza, 2008), as well as financial concerns that prevent them from participating in unpaid research (Hurtado et al., 2008). Furthermore, research has found that not identifying as a scientist also contributes to the STEM pushout (Camacho et al., 2021; Carlone and Johnson, 2007). Though there is a lack of information about preventing STEM pushout, there is an even larger deficit of knowledge about the outcomes of students at MSIs. This is a critical population since MSIs educate a disproportionate share of low-income and first-generation students and produce a significant percentage of minority graduates in STEM fields (National Academies of Sciences, Engineering, and Medicine, 2019). Moreover, this focus is future oriented since demographic shifts in the U.S. population and changes in college attendance patterns will only accentuate the significance of MSIs in higher education.

## 2 Literature review

Undergraduate research has been well-documented as a valuable experience that can enhance the odds of STEM majors completing their undergraduate education and continuing to postgraduate training in their field. Students generally perceive positive academic, professional, and personal benefits from conducting undergraduate research (Thiry et al., 2011, 2012). Longer-term experiences with faculty mentors are often viewed as more productive, leading to co-authored publications, and increased grant funding for the mentor (Morales et al., 2017). Such ideal collaborative undergraduate research experiences (UREs) promote better student learning outcomes, advance the faculty mentor’s research agenda, and make new contributions to their field (Lopatto, 2007). The outcomes of participation in UREs are particularly positive for minoritized students, where multiple studies have demonstrated their engagement in research is associated with increased persistence, higher GPAs, higher enrollment in further education and success in STEM careers (Camacho et al., 2021; Chun et al., 2022; Collins et al., 2017b; Eagan et al., 2013; Espinosa, 2011; Haeger and Fresquez, 2016; Hathaway et al., 2002; Hernandez et al., 2018; Johanson et al., 2022; Lin et al., 2024; Martinez et al., 2018; Maton et al., 2016; Monarrez et al., 2022; Simmons, 2018; Vu et al., 2023; Whittinghill et al., 2019). When URE participation is combined with near-peer mentoring, minoritized students pursuing STEM degrees tend to succeed in greater numbers as they benefit further from the practical information and strong psychosocial support they receive from the peer mentors (Trujillo et al., 2015).

While there is growing evidence of the positive impact of URE participation for all undergraduate students, including minoritized students, most of the surveys and analyses were done on a single research program in a single institution or in multiple institutions that were majority white. What is also not known is whether the undergraduate research benefits are similar across different types of research experiences. For example, many students seek out volunteer research assistantships in faculty research labs or conduct their own projects through independent study courses. Others participate in funded formal research training programs such as NSF’s Research Experiences for Undergraduates (REUs), Ronald McNair Programs or NIH training programs. In this article, we aimed to investigate the impact of undergraduate research participation on applying for graduate or professional degrees using a large consortium-wide data-set of undergraduate students across 10 public and private MSIs (Andreoli et al., 2017; Collins et al., 2017a; Estrada et al., 2017; Foroozesh et al., 2017; Kamangar et al., 2017; LaCourse et al., 2017; Richardson et al., 2017; Saetermoe et al., 2017; Taylor et al., 2017; Urizar et al., 2017; Minority Serving Institutions Program and U.S. Department of the Interior, 2015). We specifically examined whether application to graduate/professional school was associated with underrepresented minority (URM) status of undergraduate students, with their science self-efficacy scores, and their cumulative college GPA. Furthermore, we compared these associations across types of URE participation, that is, participation through formal research program (UREP), participation but not in formal program (UREnP), and no participation (No-URE), ethnicity and URM group status, gender, and biomedical major categories. This study defines biomedical major according to the standard set by the Diversity Program Consortium (2023). This study does not include data on whether the students were accepted to these graduate/professional degree programs or whether they matriculated.

## 3 Materials and methods

### 3.1 Setting

The 10 MSIs listed in Table 1 have been National Institutes of Health Building Infrastructure Leading to Diversity (BUILD) program awardees since 2014. The BUILD program provided support to institutions to support research opportunities for undergraduates as part of their comprehensive strategies to promote diversity in the National Institutes of Health (NIH) research workforce. The BUILD initiative encourages institutions to consider and implement new and creative methods to support student success. A key feature of BUILD is its flexibility, allowing applicants to develop innovative plans that address specific needs at their institutions by considering factors at the institutional, social, and individual levels. However, all BUILD awardees implemented undergraduate research programs tailored to their student populations and needs and were required to collect data on the student outcomes. In Table 1, there is information about the BUILD institutions and student demographics. Each year, ranging from 2017 to 2020, students were invited to participate in the surveys, either the HERI Annual Student Follow-Up Survey (SAFS) or the HERI College Senior Survey (CSS). Then out of the responses in that particular year, the respondents to the CSS were selected out of that group, which yields the percentage of seniors in the pool of survey respondents. For example, in 2017 27,332 students were invited at all 10 BUILD sites, 4,623 responded to the survey request and of those respondents, 874 were seniors and eligible for the CSS. Similarly, in 2018, 40,623 were invited, 4,349 responded, and 1,001 CSS responses were obtained. In 2019, 50,007 students were invited, 9,737 responded, and 2,635 were seniors and answered the CSS. Finally, in 2020, 52,082 were invited, 8,814 responded, and 1,530 were CSS respondents. Phase I of the BUILD program took place from October 2014 to June 2019 (Norris et al., 2020). Phase II started in July 2019 and will continue through June 2025. Together with the Coordination and Evaluation Center (CEC) at UCLA (McCreath et al., 2017) and the National Research Mentoring Network (NRMN) (NRMN – National Research Mentoring Network, 2024; Sorkness et al., 2017), BUILD awardees are members of the NIH Diversity Program Consortium (DPC) (DPC, 2024). The Coordination and Evaluation Center has been tasked with externally evaluating the impact of the various interventions related to the DPC’s overarching objectives in the Enhance Diversity Study (McCreath et al., 2017). All 10 BUILD institutions have implemented various forms of undergraduate research experiences, either as part of their BUILD initiatives or through other mechanisms, which may include academic year and/or summer session.

### 3.2 Study design, participants, and data collection

The current study focuses specifically on students from the MSIs listed in Table 1 who self-identified as seniors, and the impact that their participation in undergraduate research experiences (UREs) had on their application to graduate and professional programs. The study differentiates students participating in any formal program (URE-P) from those also conducting research with faculty but not as part of formal programs (URE-nP), and no research experience participation at all (no URE). However, note that this study is not differentiating BUILD program participants from students participating in other formal undergraduate research programs. BUILD program participants were defined by the CEC as students who received any type of benefit from BUILD initiatives, which could be in the form of scholarship funds (tuition, stipend), participation in BUILD training (workshops, research, courses, etc.).

Every year, starting in Spring 2017, BUILD participants and a large sample of non-BUILD sophomore, junior and senior students from all 10 institutions have been recruited to complete the SAFS. Administration of the surveys was approved by the University of California, Los Angeles, Institutional Review Board (#15–001776), and locally at each institution as required. Non-BUILD students invited to participate are sampled from biomedical and non-biomedical majors. Thus, the analysis also includes biomedical and non-biomedical majors at these MSIs, as defined by the NIH classification of majors (DPC, 2024). Students who identify themselves as seniors at the beginning of the survey are directed to take the CSS. All scales included in the HERI SAFS and CSS are validated scales and mean scores reported are computed as informed by the estimated item response theory model.

The present study focuses on four cohorts of students who completed the CSS in 2017, 2018, 2019 and 2020. The timing for first invitations to participate in the surveys varied by institution and took place between January and March of each year. The surveys were active until mid to end of July. To enhance the response rates, a pre-notification email was sent to students from each institution by institutionally selected individuals (influencers) to invite participation in the study. Three to four email reminders, distributed over the period the survey link was active, were also sent to non-respondents (Manfreda et al., 2008). Participants were awarded with a $25 gift card to incentivize survey completion. A total of 6,040 students completed the CSS, which corresponds to 21.94% of all students completing the combined SAFS and CSS (27,523). The CSS contains sociodemographic information as well as several questions on undergraduate research experience, whether this was through a formal program or independently sought and whether the student applied to graduate or professional studies. To address the research questions, we selected several items from the CSS, and a full description of the questions is included in Appendix A.

### 3.3 Statistical analysis

Following de-identified data import, cleaning, and validation, missing value information was analyzed to identify any patterns. The cleaning process consisted of checking all variable ranges and values for accuracy and checking for missing values not indicated by an appropriate missing value label when needed and validation of the scales to the assumed construct being measured. A missing values map identified where data were missing and found no strong pattern associated with missing values. A multiple imputation model using chained equations was then utilized to insert model-based values and, thereby, reduce bias and improve the representativeness of research results (van Buuren and Groothuis-Oudshoorn, 2011). Following data imputation, transformations were made to variables to ensure the appropriate formatting. For example, factors were informatively coded, levels of factor collapsed when necessary, and references for factors were set. Finally, a subset of the predictor variables was selected that were associated with inclusion in an organized research program but not associated with the outcome of interest (e.g., graduate/professional school plans/application). The variables selected in this manner were (1) number of months in mentored research (1 = 0 months, 2 = 1–3 months, 3 = 4–6 months, 4 = 7–12 months, 5 = 13–24 months, 6 = 25+) status as an underrepresented minority (1 = URM, 0 = not URM); (2) college cumulative GPA (A, B, C, D/F); (3) biomedical major indicator (1 = biomedical major, 0 = not biomedical major); (4) science self-efficacy sum score; and (5) gender identity (1 = male, 2 = female, 3 = non-binary, trans, other). For the purposes of this study, a biomedical major refers to basic biomedical sciences (including behavioral and social sciences) that can lead to the pursuit of “a Ph.D. in a field that deals with the biological mechanisms that are ultimately related to human health” (National Research Council, Policy, Global Affairs, Board on Higher Education, Committee to Study the National Needs for Biomedical, and Clinical Research Personnel, 2011).

Using this set of variables, propensity scores were calculated for inclusion in an organized undergraduate research program using boosted logistic regression models and the maximum of the Kolmogorov–Smirnov statistic as a stopping rule (Cefalu et al., 2024). The weights were extracted from the propensity scoring model and inverse probability weighting using the propensity scores were applied to the generalized linear models depicting associations between graduation plans and science self-efficacy, underrepresented minority status, and college GPA. Using the weights resulting from propensity scores reduces the impact of selection bias on results pertaining to URE inclusion. For example, if the data were not weighed in this manner, there would be validity to the claim that all associations are partially due to systematic differences between the URE and non URE groups. This could reasonably occur if higher performing students self-select to the URE programs. All summary tables, models reported, and analysis use the imputed and propensity score weighted data.

To investigate the three research questions associated with graduate/professional school plans, a single multivariate binary logistic regression model was utilized to predict graduate/professional school plans/application. Subset analysis, using participation in an organized research program, was performed for assessing associations between graduate/professional school plans and the following primary explanatory variables: underrepresented minority status, science self-efficacy, and college GPA. Other control variates were included, such as gender and number of months in mentored research. Variables such as college affiliation and age, among others, were considered as control variates in the model, but either lacked appropriate variation to be suitable predictors or were not associated with the predictor variable set or outcome variable. The models were built progressively, starting with a baseline model of all main effects and then fitting interactions based on improvement to model fit. Model results are summarized using odds ratios where, if the interval estimate contains 1, then there is no implied association. Finally, data visualizations, including profile plots and forest plots summarize model results.

As a last stage in the analysis, a dose response analysis was conducted on all biomedical major students. In this analysis, the students majoring in a biomedical relevant discipline were separated into majors with a social science focus and those with a natural science focus (National Research Council, Policy, Global Affairs, Board on Higher Education, Committee to Study the National Needs for Biomedical, and Clinical Research Personnel, 2011). The overall binary logistic regression model was then used to create inverse predictions and estimate the number of months needed to ensure that students had at least a 70% probability of application to graduate or professional school. This analysis was conducted on the full adjusted model and made use of the variable recording the number of months in mentored research.

## 4 Results

### 4.1 Descriptive statistics results

Table 2 presents demographic information on the research participants in the study. Note that there is evidence of bivariate associations between plans for graduate and professional school, participation in mentored research, and the demographic features of race/ethnicity and gender as indicated by the *p*-values testing for associations between the groups and demographic characteristics.

In Table 3, exploratory analysis results are presented on the major study outcomes for research participants in the study. The data are divided using the outcome variable, application or no application to a graduate or professional school, and participation in research either as part of an organized undergraduate research experience program (URE-P), participation in research but not as part of an organized program (URE-nP) or no research participation (no URE). The table provides summary statistics for each combined application and research program group, along with Fisher’s exact tests of association for these factors performed using simulated p-values. Note that these major cohort characteristics differ between the six groups and provide evidence of major factors influencing the decision to apply to a graduate or professional school.

**FIGURE 1:**
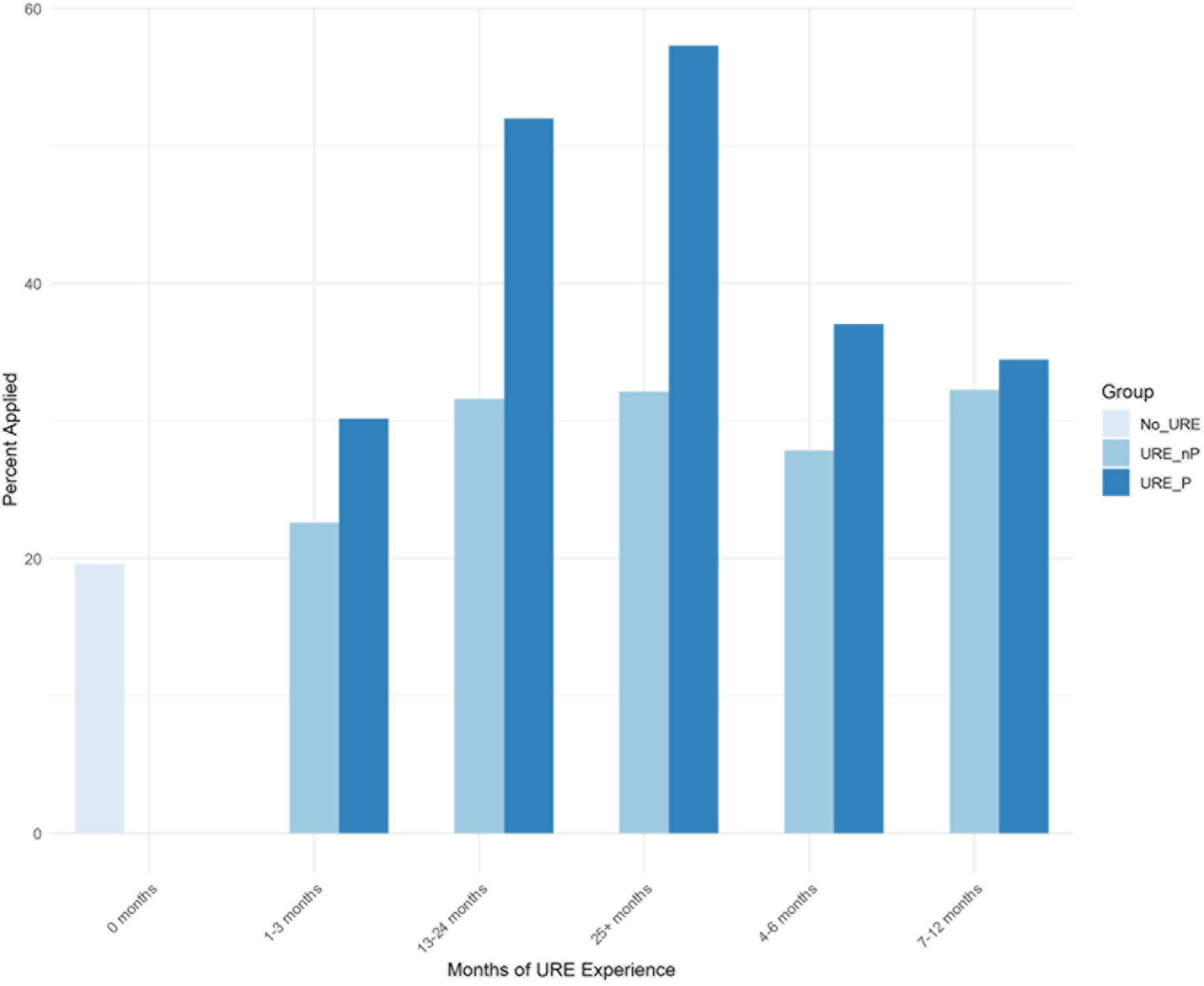
Dosage effect of participation in URE formal program vs. no program on application to advanced degrees.

Exploratory analysis reveals the following important themes directly emerged from the data in Table 3. First, Figure 1 shows a comparison between undergraduate researchers who participated in formal URE programs vs. those active in research but not in programs with respect to the time they did research with faculty and their application or not to advanced degree programs. These data reveal that, overall, students in programs apply at higher rates than those not in programs. Students in programs who participate for more than one full year (i.e., 13–24 months and 25 + months) apply at much higher rates (52 and 57%, respectively) than those not in programs but did research for the same amount of time (31 and 32% respectively). Furthermore, students who participate in formal URE programs for 13 months or more also apply at higher rates (54% on average) than those whose URE program experience is shorter than 13 months (on average 27% applied).

**FIGURE 2:**
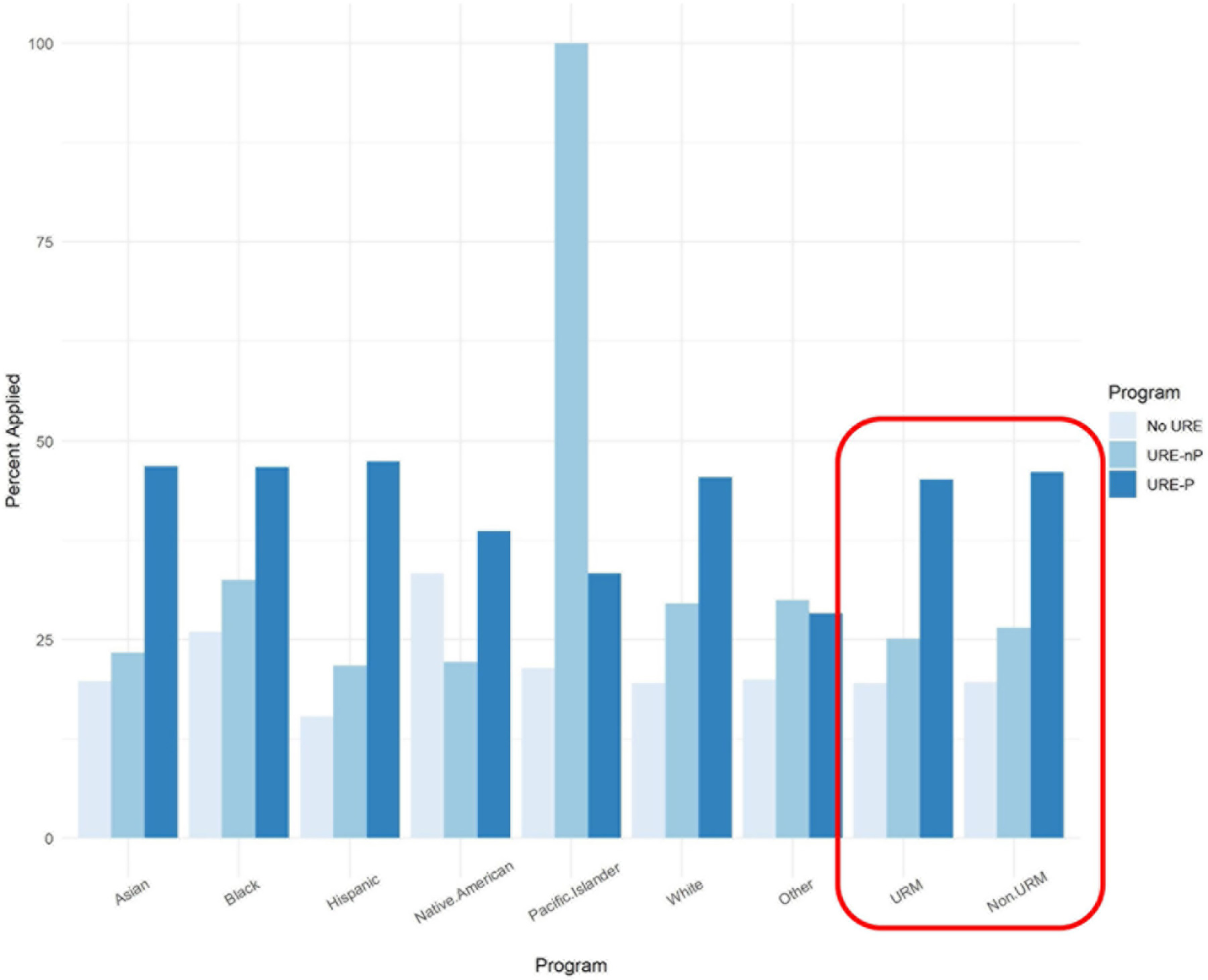
Research participation in URE programs vs. not in programs in comparison with no research participation on application to advanced degree programs by URM status.

The second theme that emerged from the data in Table 3, which addresses research question one (application to graduate/professional school and URM status), is the effect that participation in formal URE programs has on the application to advanced degrees by URM students surveyed at the 10 institutions involved in this study. In looking at the last two sets of bars in Figure 2, it appears that, in general, URM students conducting research as part of formal programs are applying to enter graduate and professional degree programs at higher rates than URMs not in programs and at much higher rates than URMs who do not participate in research (Zero months). These percentages are largely due to the numbers of Black and Hispanic respondents participating in programs and to a smaller extent to Native Americans and “Other,” since the actual numbers of Pacific Islanders in URE-P (*N* = 9) and URE-nP (*N* = 1) are too small to reach individual conclusions about these groups. However, in aggregate, URM researchers in programs (*N* = 518) apply at similar rates as non-URMs in programs (*N* = 684), that is 45 and 46%, respectively. Similarly, URM researchers who did not participate in formal programs (*N* = 354) apply at a similar rate as their non-URM counterparts (*N* = 457), that is 25 and 26%, respectively.

**FIGURE 3:**
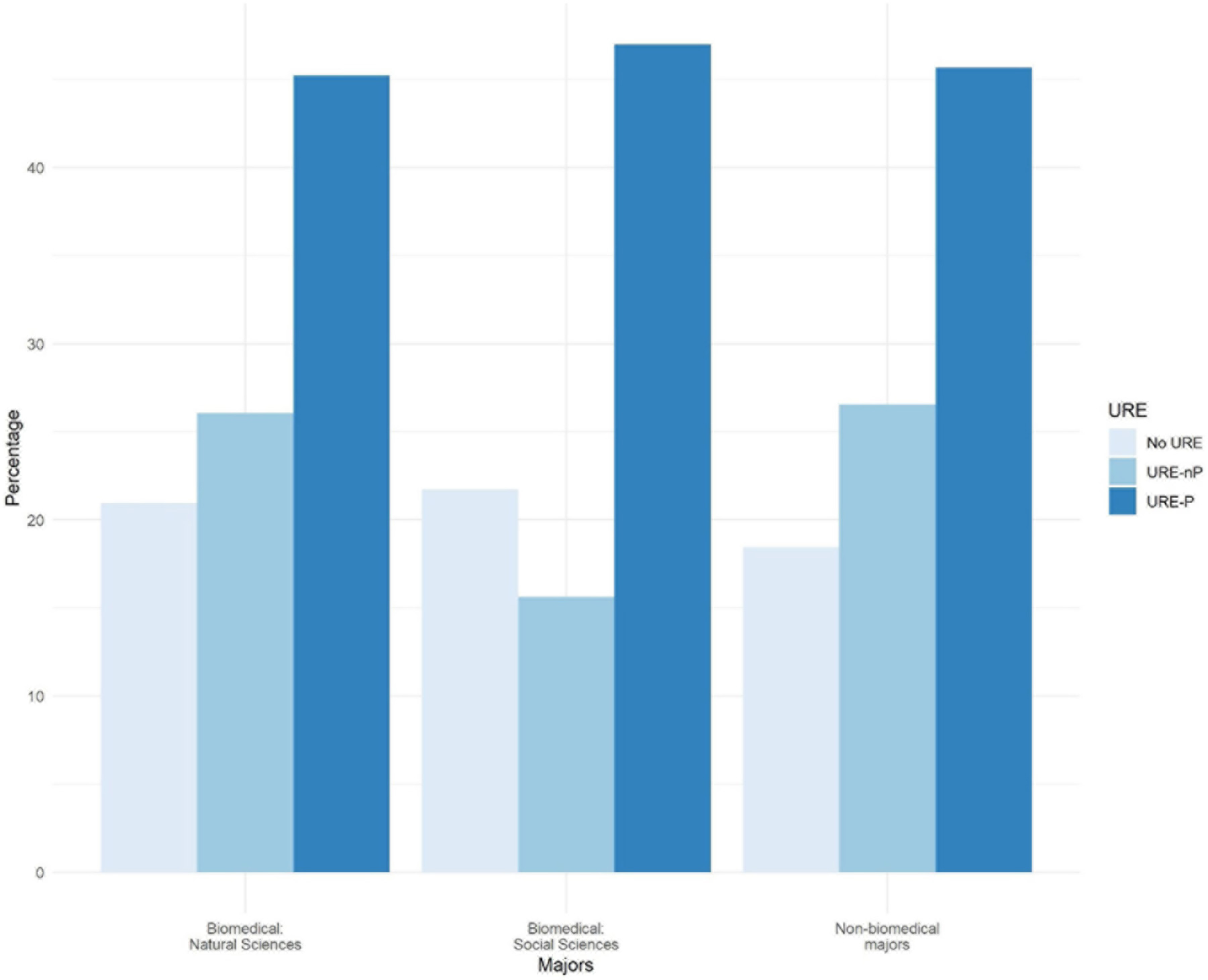
Participation in URE-P, URE-nP on application to advanced degree programs by type of biomedical major vs. no participation.

A third type of association was found between the type of biomedical major, in combination with participation or not in UREs, and the rate of application to advanced degree programs. As shown in Figure 3, undergraduate researchers from all majors in formal programs pursuing natural, social sciences as well as non-biomedical majors, apply at about the same rate (45, 47.0, and 46% respectively). Students in all majors who participate in formal research programs apply at much higher rates as students not in formal programs. Although the pattern is positive, a word of caution is warranted with respect to social sciences majors in URE-nP, as the raw numbers of respondents are too small to reach definitive conclusions.

**FIGURE 4:**
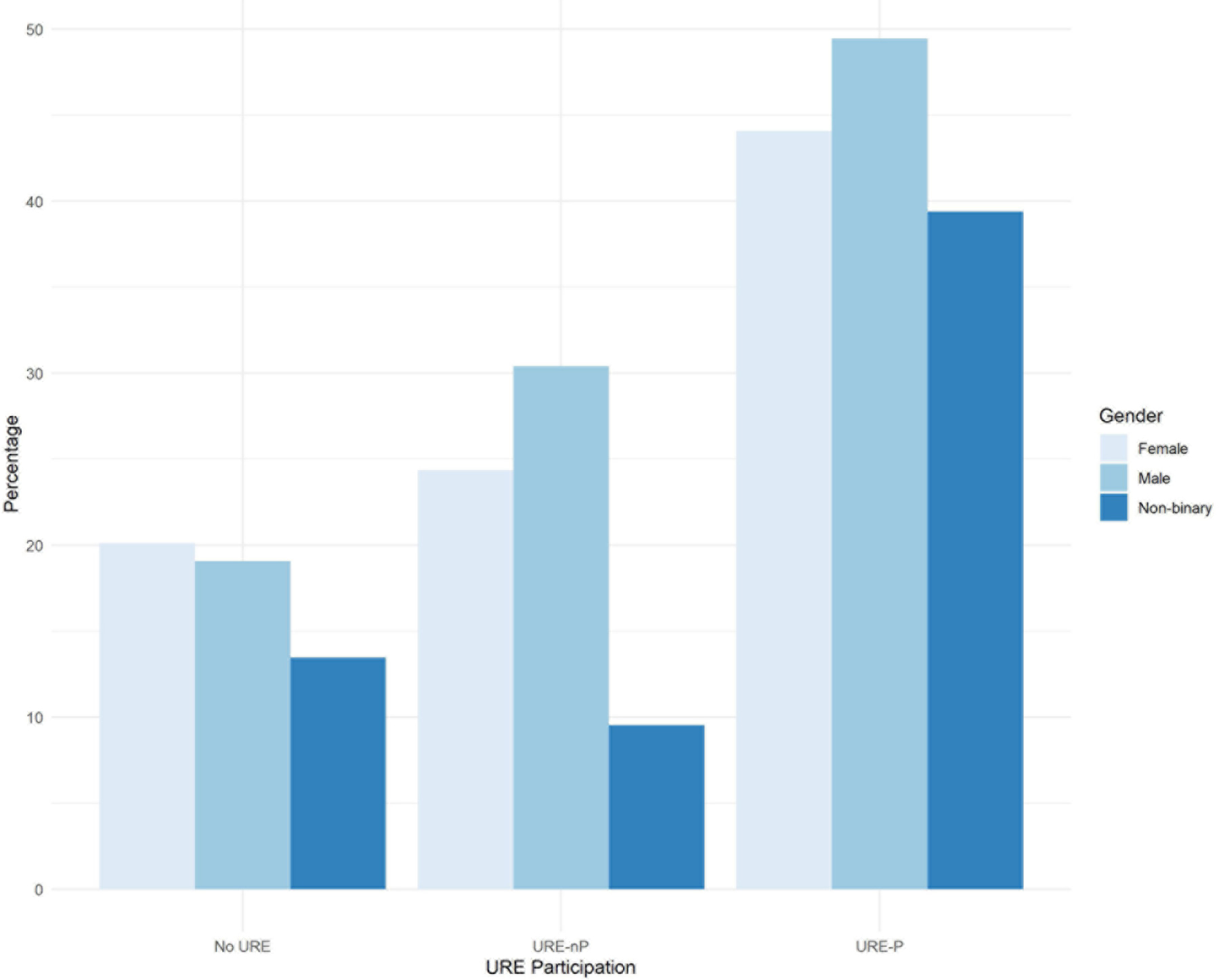
Percentages of application to graduate/professional programs by gender and research participation.

Focusing on sex raw numbers (see Table 2), it is notable that it appears as if twice as many females than males participated in research experiences (*N* = 1,197 vs. *N* = 560, respectively), counting both URE-Ps and URE-nPs. However, a word of caution is warranted here, as those raw numbers can be attributed to more females responding to the survey than males, 2,310 vs. 1,095, respectively. The proportion of male vs. female students at the 10 institutions is 45% vs. 55%, respectively. Nevertheless, as shown in Figure 4, the proportion of applications to grad/professional programs is larger in males than females for URE-P and URE-nP groups. Non-binary students had lower rates of application overall compared to both males and females.

**FIGURE 5:**
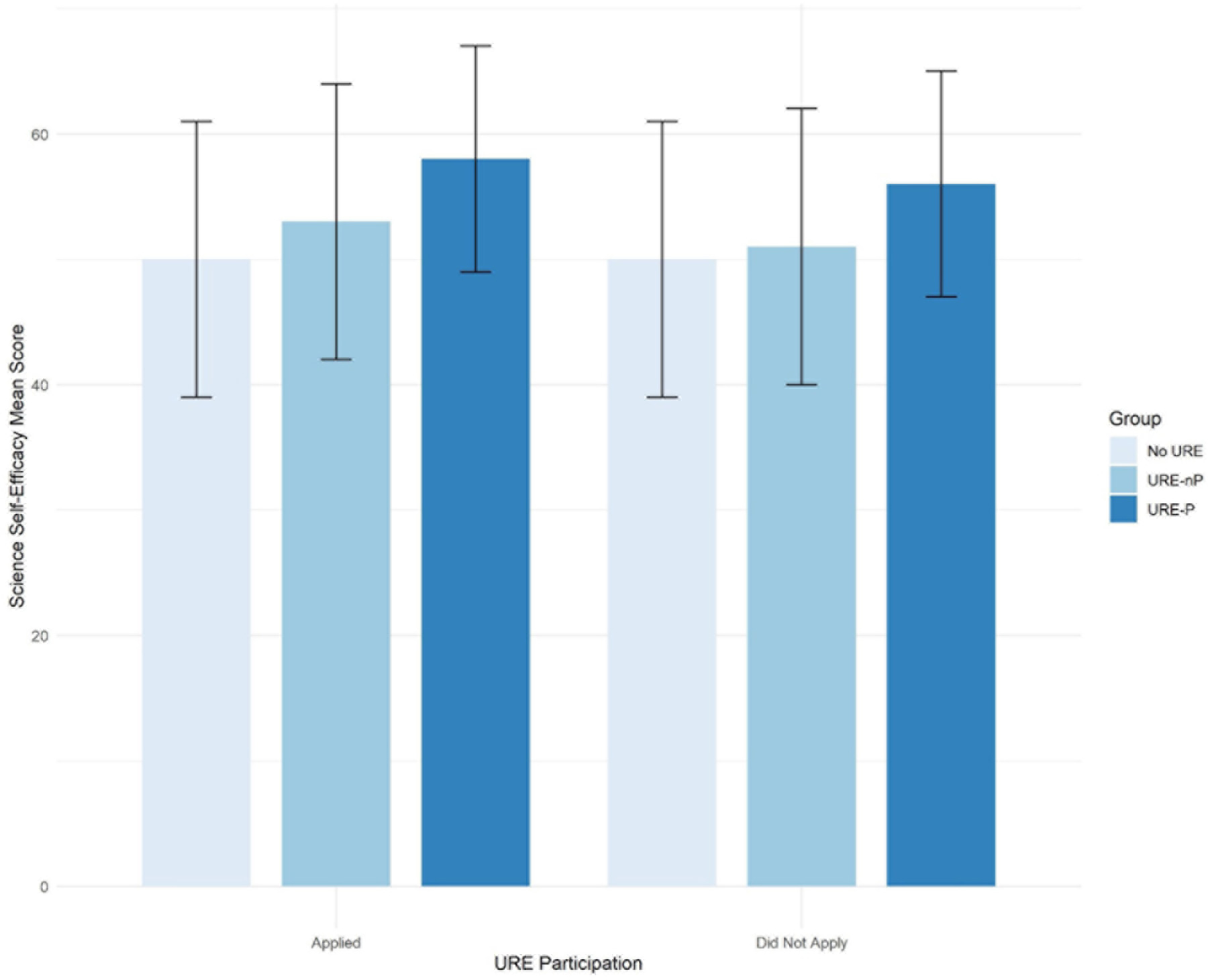
Science self-efficacy sum scores of students who applied to graduate/professional programs.

To answer research question #2 (association between application to graduate/professional school and science self-efficacy), a self-efficacy sum score was obtained for each participant’s responses to the self-efficacy construct in the survey (see Supplementary data). Mean scores are reported in Table 3 and shown in the bar chart in Figure 5. For students who applied to advanced degrees, the science self-efficacy sum score is slightly higher for URE-P participants than for URE-nP participants, which in turn is slightly higher than those who did not participate in research at all.

**FIGURE 6:**
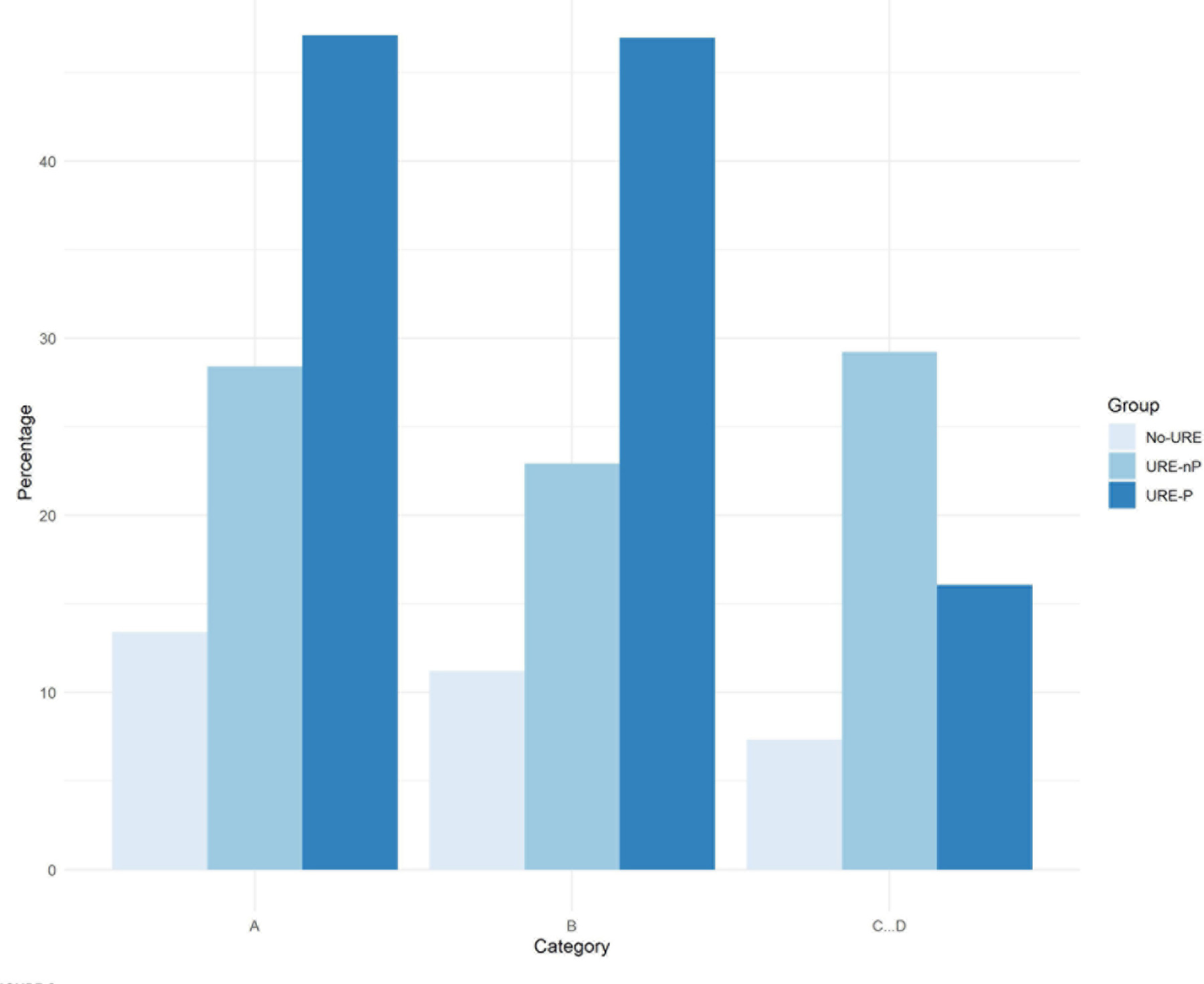
Proportion of application to advanced degrees for students in URE-P vs. students UREnP and students who never participated in an URE grouped by average GPA.

With respect to research question #3, which refers to the effect of overall GPA on application to advanced degrees, Figure 6 shows a graphical representation, in terms of percentages, of results presented in Table 3. As expected, URE-P students with overall GPAs of A and B apply to advanced degrees at much higher rates than UREnP and noURE students (around 22 and 35 percentage points, respectively). Interestingly, researchers not in formal programs with an overall GPA of C apply to advanced degrees at higher rates than those in programs, and at much higher rates than those who did not participate in any form of mentored research experience. The data suggest that the largest effect of research program participation is on students with GPAs of A and B, although for those with GPA of C there is still a benefit in comparison with no URE participation. This increased proportion of students applying to graduate programs in the C GPA range is probably due to the use of GPA thresholds for admission and retention in organized research programs at most institutions. Further analysis to verify is presented below.

### 4.2 Model-based results

An abridged presentation of model results is provided in Table 4, and a full report of the model results appears in the Appendix A. An Rao-Scott likelihood ratio test (LRT) test provides evidence that URE participation impacts graduate and professional school application (LRT = 322.24, *p* ≤ 0.0001). The full model in the Appendix A provides the original estimated model from which the odds ratios were computed using a linear combination of the model parameters, whereas Table 4 provides model-based odds ratio estimates for each research question (RQ 1, 2 and 3) associated pointwise 95% confidence interval estimates. Results indicating statistical significance (where the odds ratio interval estimator does not include 1) are statistically significant results.

#### RQ 1: are graduate/professional school plans/application associated with URM status?

**FIGURE 7:**
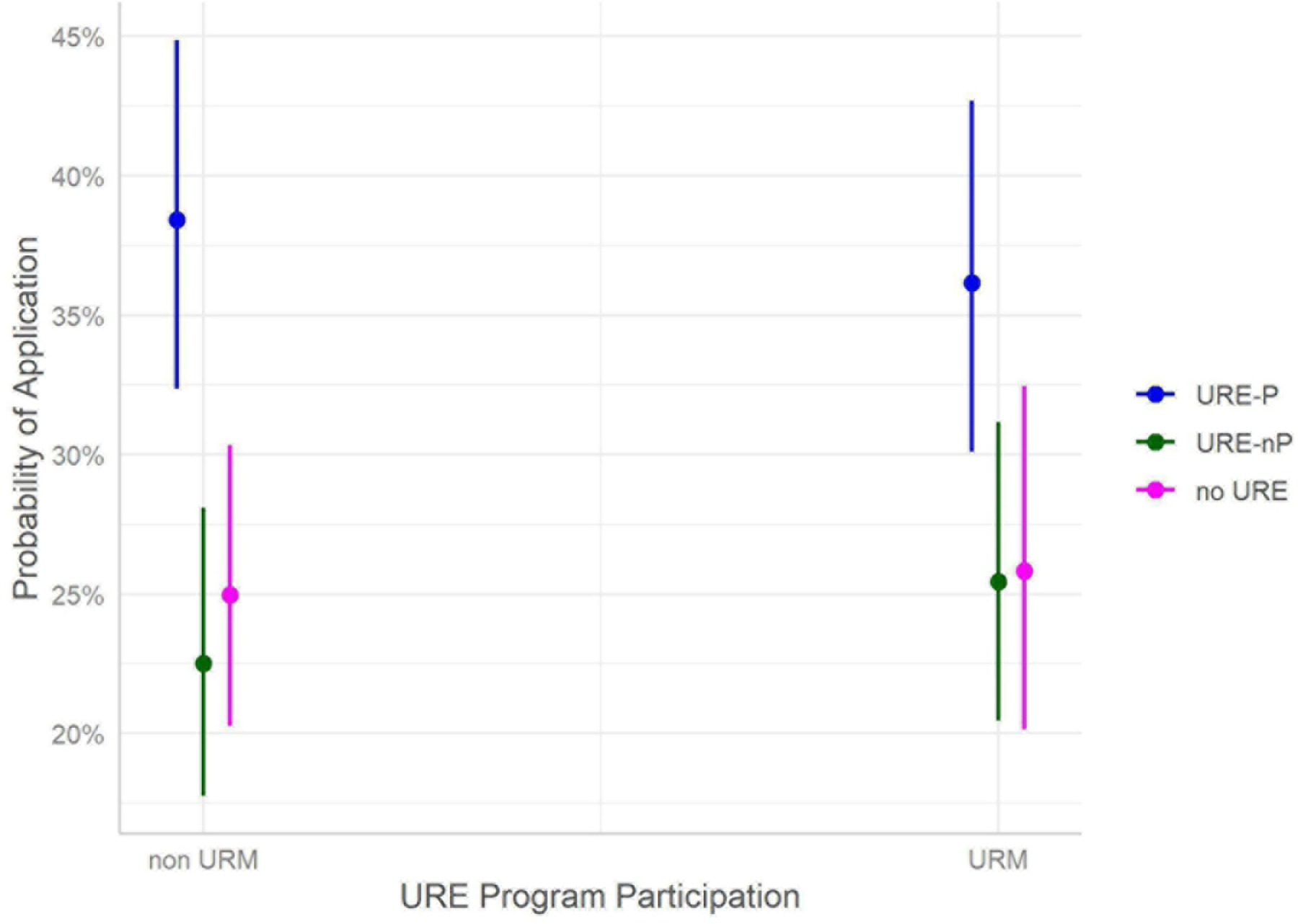
URM status and association with graduate/professional school application, subset by URE participation.

The results indicating impact of URM status suggests no observed difference between URM and non-URM cohorts across any of the URE groups [URE-P OR: 0.91 (0.72, 1.15); URE-nP OR: 1.175 (0.88, 1.50); no URE OR: 1.05 (0.78, 1.42)]. This implies that URM students achieve similar outcomes regarding application to graduate or professional school when compared to their non-URM cohorts within each level of undergraduate research experience. Figure 7 provides a visualization of the probability of application for URM and non-URM cohorts in or not in a URE. Note that there is an increase in odds of applications for URE-P participants, but the difference between URM and non-URM cohorts is not statistically significant. Note that, for the surveyed students, 57% of the URM students participated in research (URE-P and URE-nP), while only 45% of non-URM students participated in research as reported in Table 2.

#### RQ 2: are graduate/professional school plans/application associated with science self-efficacy?

**FIGURE 8:**
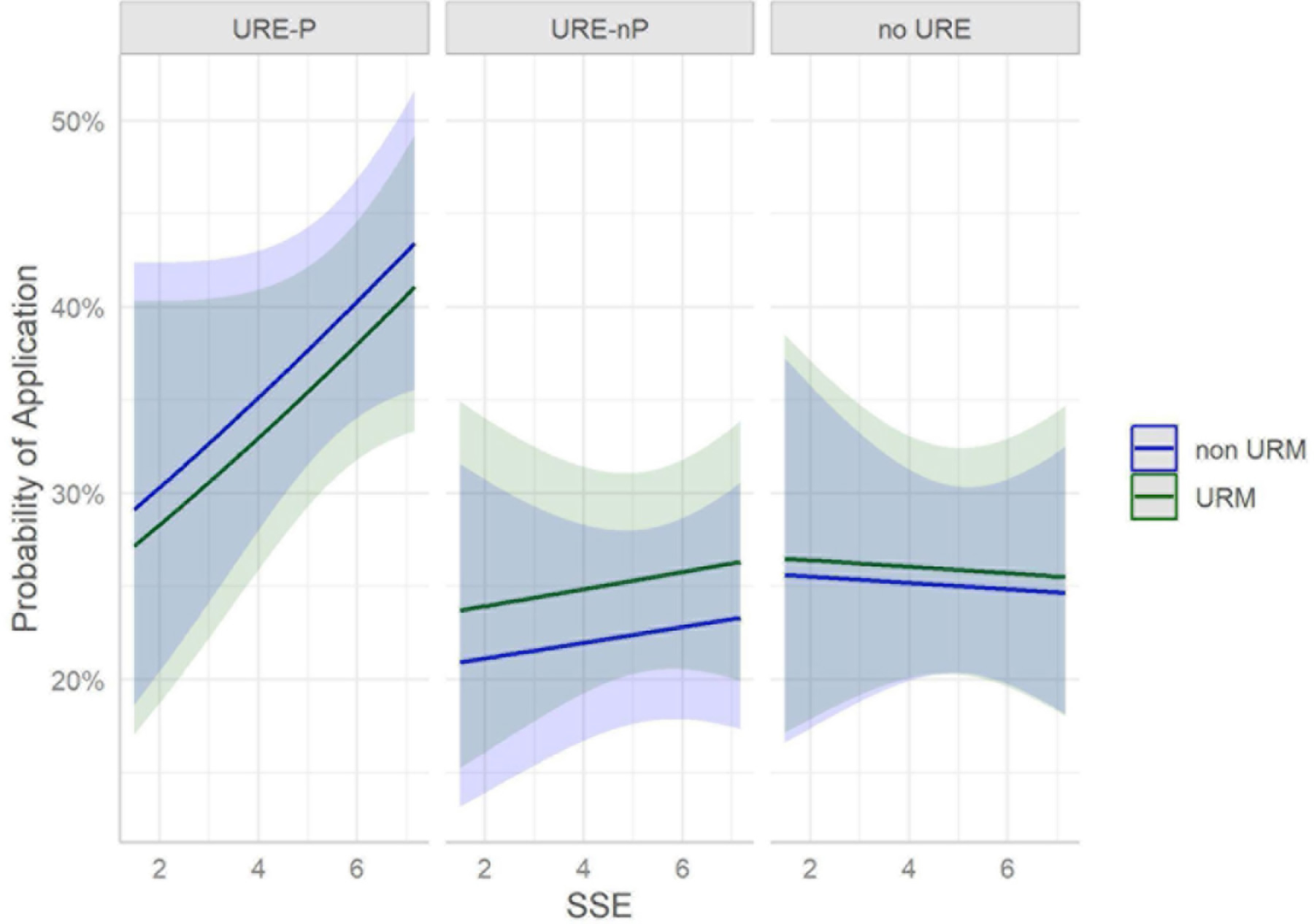
Science Self-efficacy Scores and association with graduate/professional school application, subset by URE participation.

The effect of science self-efficacy (SSE) on graduate/professional school plans (application, no application) varies by URE status. When students are involved in UREs with formal programs, there is a positive effect associated with a 10 unit increase in SSE. That is, a 10 unit increase in SSE increases the odds of application by about 12% on average [OR(URE-P) = 1.12; CI: 0.98, 1.27] with only marginal significance in URE-P populations. Figure 8 visualizes the impact of SSE on application rates among the URE-P trainees. The bars indicate 95% confidence intervals on the probability of application. Additionally, note the negligible difference in impact of SSE on application rates among URM and non-URM students. In contrast, non-program URE cohorts experienced no change in odds of application vs. no application with a 10 unit increase in SSE [OR(URE-nP) = 1.03; CI: 0.91, 1.16]. Among students in no URE program, there is also no impact on SSE change in either direction [OR(no URE) = 0.99 (0.87, 1.13)]. Figure 8 displays this interactive effect of science self-efficacy and URE participation on graduate/professional school applications. It also includes the model derived probability of application separated by URM status, providing evidence that URM status has very little impact on participation. Note that there is no difference in impact of SSE on probability of application for URM and non-URM cohorts in each URE group.

#### RQ 3: are graduate/professional school plans/applications associated with cumulative college GPA?

This research question is answered by comparing odds of application to graduate/professional school for those with one level of cumulative GPA vs. another level. The levels considered are those with A, B, or C, D equivalent GPAs, as reported by survey respondents. Analysis was performed with an interaction between URM status so the impact of GPA on probability of application is differentiated between URM and non-URM cohorts.

**FIGURE 9:**
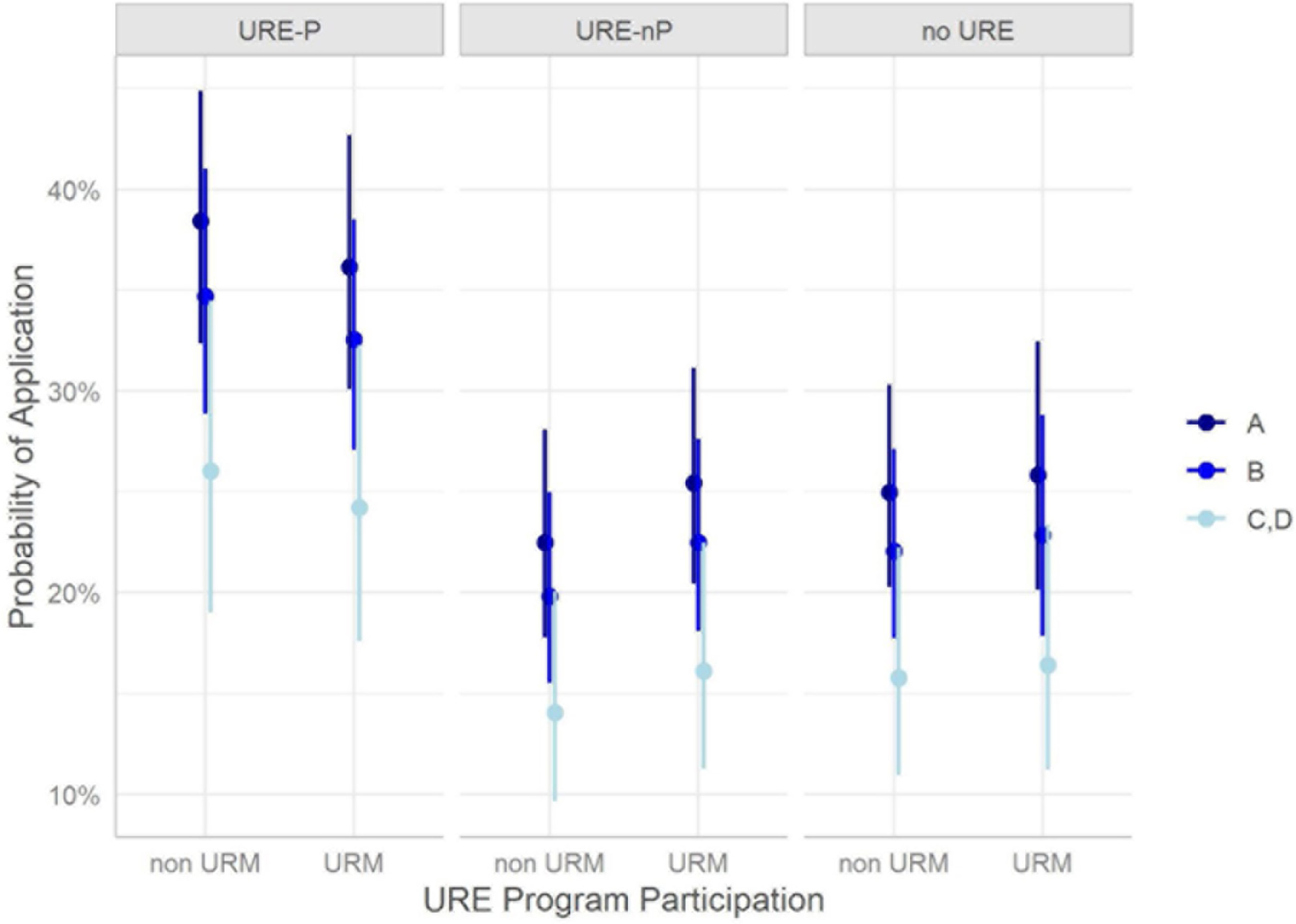
GPA and association with graduate/professional school application, subset by URE participation.

When comparing those with an A vs. B equivalent GPA, for students in the URE-P cohort, there no significant association between having an A vs. B average and application to graduate/professional school for URM and non-URM populations [OR(URE-P, non-URM) = 1.15 (0.80, 1.66); OR(URE-P, URM) = 1.07 (0.77, 1.50)]. Similarly, for students engaged in URE with no program, there was no detected association [OR(URE-nP, non-URM) = 1.33 (0.89, 1.97); OR(URE-nP, URM) = 1.24 (0.85, 1.79)]. For the no URE students, there is only a marginal positive association for A vs. B students among non-URM students [OR(no URE, non-URM) = 1.50 (1.00, 2.24); OR(no URE, URM) = 1.40 (0.91, 2.15)]. When comparing A vs. C, D students, there is a strong positive increase in odds for URE-P students [OR(URE-P, non-URM) = 4.79 (1.85, 11.41); OR(URE-P, URM) = 5.11 (2.08, 12.57)] and no association detected for other URE groups. Finally, for B vs. C, D GPA comparisons, there is an increase in odds for URE-P cohorts only [OR(URE-P, non-URM) = 4.15 (1.58, 10.89); OR(URE-P, URM) = 4.76 (1.94, 11.68)]. Figure 9 visually depicts the odds of application for the URE and non-URE cohorts over different GPA groupings by depicting the mean response and associated 95% confidence bands. In this figure, the results are further subdivided by URM status. In general, there are consistently narrower gaps between URM and non-URM student probabilities by program type. However, students in the URE-nP and no URE groups have lower odds of application overall. Since all GPA results are self-reported, some caution should be taken with all interpretations.

##### 4.2.1 Dosage analysis

As a follow up to the three primary research questions, we investigated the relationship between the amount of time spent in mentored research (research dosage) and application status for the subpopulation of biomedical major’s students. Due to evidence in the literature about differences in research dosage effect for students in social and behavioral sciences vs. laboratory sciences, we conducted the analysis separately for these two cohorts (Adedokun et al., 2013). Using the full model, we use inverse predictions to estimate the needed research dosage that ensures that at least 70% of the students apply for graduate or professional school. In the survey, the students indicated the number of months of mentored research with levels: 1 = 0 months, 2 = 1–3 months, 3 = 4–6 months, 4 = 7–12 months, 5 = 13–24 months, and 6 = 25 + months. This assumes a linear impact of research months on the probability of application. While this is a substantial assumption, we feel it is substantiated by the data and is a reasonable approach.

**FIGURE 10:**
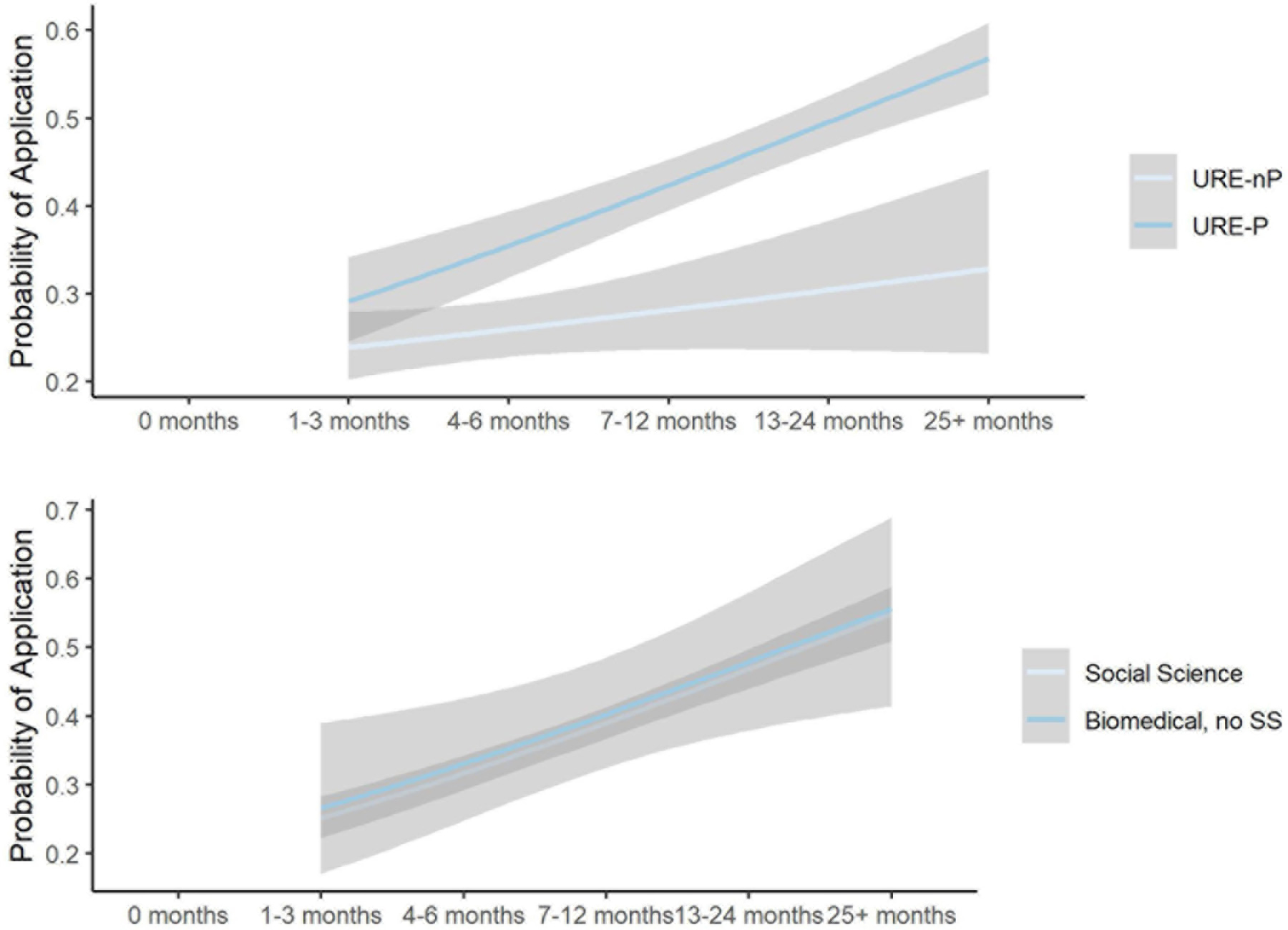
Mentored research months impact on graduate/professional school application, subset by URE participation.

Per the descriptive analysis, the data suggest that 12 + months is the ideal dosage of participation in URE programs at MSIs to elicit more interest in applying to advanced degrees. These results also confirm conclusions from a prior study (Hernandez et al., 2018) involving fewer students (577-all URM) from 29 different institutions (some non-MSIs) and majoring in the natural sciences and engineering. A formal dose response analysis also confirms this and specifies dosage by major (biomedical major and social science major). In this further analysis, we omitted non URE students from the analysis since they did not participate in research hours. Note that the models extrapolate beyond the range of the data, but we are presenting these results with a major caveat. These are predictive of needed research and all we can assume about inverse predictions that are >60% is that at least 25 months of research is required. All model-based results about the dosage of research months needed to achieve a particular probability of application are presented in Figure 10. Regarding URE groups, results indicate that URE-P students require less time in mentored research than URE-nP students. There appears to be little difference between biomedical-natural science and biomedical-social science majors. However, to attain higher probabilities of application, a longer research experience is needed for non-social science biomedical majors.

## 5 Discussion

In this study, we addressed the effects of participation in undergraduate research on student application to advanced degree programs. We collected responses to the HERI Annual Student Follow-Up Survey (SAFS) from undergraduate seniors (CSS) at 10 schools that participate in the NIH-funded BUILD program (Norris et al., 2020), and are part of the Diversity Program Consortium of the NIH that focuses on innovative undergraduate training to increase the diversity of the biomedical and behavioral health research workforce (Valantine and Collins, 2015). As part of this program, we pooled data from all undergraduate respondents to the survey at those institutions, whether they were undergraduate researchers (BUILD and non-BUILD) or not (Hallmarks of Success, 2025). Responding students were divided into three groups, based on their reported level and type of research activities: (1) Those who participated in research as part of a formal program (URE-P); (2) Those who participated in undergraduate research but were not part of a formal program (URE-nP); and (3) Those who did not report any participation in undergraduate research experiences (no URE). This study design allowed us to examine the impact of participation in the two modalities of undergraduate research experiences, formal programs and no program, across a broad range of different research experiences at different campuses. For each group, we assessed if participation in undergraduate research or the modality of the research experience increases applications to advanced degrees. We further assessed whether likelihood of applications is associated with URM status, science self-efficacy, or cumulative self-reported college GPA.

Many efforts and programs exist to increase the participation of historically underrepresented groups in science. In the natural sciences fields, many of these programs focus on engaging students from diverse backgrounds in high-impact activities such as undergraduate research (Committee on Closing the Equity Gap: Securing Our STEM Education and Workforce Readiness Infrastructure in the Nation’s Minority Serving Institutions, Board on Higher Education and Workforce, Policy and Global Affairs, and National Academies of Sciences, Engineering, and Medicine, 2019; Committee on Strengthening Research Experiences for Undergraduate STEM Students, Board on Science Education, Division of Behavioral and Social Sciences and Education, Board on Life Sciences, Division on Earth and Life Studies, and National Academies of Sciences, Engineering, and Medicine, 2017). The long-term impact of these programs is often not known without longitudinal studies. The reason is that the impact cannot be measured if the student is still in their undergraduate training program and may choose to engage in post-baccalaureate training prior to applying for professional and graduate programs. In this current study, we were able to come closer to an assessment of impact by examining cross sectional surveys over a multi-year time period to begin to understand the relationship between undergraduate research and advance degree program applications.

The major findings of our study indicate that engagement in a broad array of undergraduate research environments is an effective intervention to increase application to graduate school by participating students compared to those not engaged in undergraduate research. Moreover, participation in formal programs further increases application to graduate school, especially for those students who participated in programs for longer than 12 months (Figure 1). Among our respondents, students from underrepresented minorities applied to graduate programs at similar rates as non-URM students regardless of participation in undergraduate research or formal programs (Figures 2, 7). Similarly, for students participating in undergraduate research, their declared major area of study (natural sciences vs. behavioral and social sciences vs. non-biomedical) did not significantly influence application rates (Figure 3). The percentage of male students who participated in undergraduate research (URE-P and URE-nP) and applied to graduate and professional programs is larger than that of females or non-binary students, although more female students responded to our surveys (Figure 4 and Table 2). The relationship between science self-efficacy and application rates was not significant for any group at *p* < 0.05 but showed positive trends for students participating in undergraduate research and no trend for those not involved in research (Figures 5, 8 and Table 4). The participation in research impacting rates of applying to graduate school and the positive impact of dosage is in agreement with previous studies which looked at the impact of time spent on a particular research project (Colclasure et al., 2024; Hernandez et al., 2018; Shaffer et al., 2014) and the overall number of unique research experiences (Merkle et al., 2023). In both cases, more time spent and higher number of research experiences is associated with positive overall impact for the students. Our findings here further support the notion that not only is participation in undergraduate research impactful on self-reported application, but that more time spent on research enhances application to graduate school. In other words, longer research engagement seems to be better for students.

Grades were associated with application rates only for those students in formal undergraduate research programs, but not with those who either conduct research independently of such programs or report no research engagement at all. Students in formal programs that have a self-reported GPA of A or B are much more likely to apply for graduate or professional programs than those earning a C grade or lower. We note that most graduate programs require a minimum 3.0 GPA for screening purposes (Walters et al., 2022). No difference in association between GPA and application status was detected between students from underrepresented minority groups and non-URM students. We do note the suggestion of a possible negative influence of formal programs on the propensity of C&D students to submit applications (Figure 6, C&D). Due to study limitations, we cannot distinguish between the varying motivations for impact of URE programs on C&D students, but can simply report the empirical results.

The finding that science-self efficacy is only marginally associated with the application to advanced programs, if at all, should be a cautionary note when using this metric as a measure of the success of programs aimed at increasing participation in professional scientific fields that require advanced academic training. While the need for short and mid-term correlates to long-term effects on student success is important, our data indicate that these effects need to be carefully evaluated. The reasons why science self-efficacy was not closely associated with application to advanced degree programs is not clear based on this study. In previous studies, science self-efficacy was associated with career trajectory (Carpi et al., 2017; Livinƫi et al., 2021); other hallmarks used as proxies for program success should be carefully evaluated based on our findings regarding the relationship (or lack thereof) between science self-efficacy and application to advanced programs. The results in this study may differ due to the inclusion of non-biomedical major students in the analysis or due to the impact of unaccounted for lurking or confounding variables in past studies that can explain the association.

Our results further strengthen the findings from other studies that participation in undergraduate research experiences, especially as part of a formal program, is a high-impact practice to support undergraduate students, including those from underrepresented minorities, on their path to advanced degrees (Goodwin et al., 2024). This interpretation of our findings should serve as encouragement for educators, funding agencies, and policy makers to continue to support, implement and expand such programs. The finding that students from historically underrepresented groups applied to advanced degree programs at similar rates compared to those from well-represented groups is encouraging, as recruitment into these programs can, therefore, serve as a strategy to improve outcomes for all students while closing gaps in representation among these groups in professional settings. Programs like BUILD and others like it can provide structure and support for undergraduate research programs, helping to remove barriers of entry and support students within these programs (Allen-Ramdial and Campbell, 2014).

Limitations of this study include that it is purely based on survey responses rather than application and ultimate enrollment in graduate or professional programs. Survey responses can create selection bias as the respondents to the survey are unlikely to be fully representative of the total student body that we intended to study.

## 6 Conclusion

This study’s findings indicate that participation in undergraduate research, particularly within a formal undergraduate research program, significantly increases the probability of students applying to graduate programs. This positive impact is further enhanced when students participate in these programs for more than 12 months. Notably, URM students apply to graduate programs at similar rates as their non-URM peers, regardless of their involvement in undergraduate research or formal programs. This suggests that recruiting students into undergraduate research programs can effectively promote academic advancement for all students while simultaneously addressing representation gaps in professional fields.

Future research should continue to explore the intersectionality of undergraduate research experiences with other known factors related to graduate or professional school application. This could identify combinations of factors that have a magnified impact on students’ probability of application. Other future research should comparatively analyze the probability of graduate and professional school application vs. enrollment. This could provide insight into how to support students who have applied to graduate or professional school programs and provide a boost to enable them to enroll once admitted to programs. All future research should also continue to focus on how to include underrepresented students while also supporting all student populations aspiring for graduate program enrollment in the biomedical sciences.

## Supplementary Material

Appendix A Supplementary Table 1

The Supplementary material for this article can be found online at: https://www.frontiersin.org/articles/10.3389/feduc.2025.1589105/full#supplementary-material

## Figures and Tables

**TABLE 1:** Participating BUILD institutions.

BUILD institution*	Institution type**	% Pell grant recipients 2017†
CSULB	Public	54%
CSUN	Public	57%
MSU	Public	55%
PSU	Public	39%
SFSU	Public	55%
UAF	Public	24%
UDM	Private	32%
UMBC	Public	30%
UTEP	Public	62%
XULA	Private	17%

* CSULB, California State University - Long Beach; CSUN, California State University - Northridge; MSU, Morgan State University; PSU, Portland State University; SFSU, San Francisco State University; UAF, University of Alaska - Fairbanks; UDM, University of Detroit - Mercy; UMBC, University of Maryland - Baltimore County; UTEP, The University of Texas at El Paso; XULA, Xavier University – Louisiana.

† U.S. Department of Education, Distribution of Federal Pell Grant Program Funds by Institution, https://www2.ed.gov/finaid/prof/resources/data/pell-institution.html.

**TABLE 2:** Demographic characteristics of study participants by URE inclusion and graduate or professional school plans.

Characteristic	Applied to graduate or professional schools			Did not apply to graduate or professional schools			*p*-values^2^
	URE-P*	URE-nP**	No URE***	URE-P	URE-nP	No URE	
	*N* = 548 (100%)^1^	*N* = 157 (100%)^1^	*N* = 333 (100%)^1^	*N* = 654 (100%)^1^	*N* = 452 (100%)^1^	*N* = 1,367 (100%)^1^	
Race/Ethnicity							<0.001
Not URM (White or Asian)	239 (44%)	75 (48%)	191 (57%)	279 (43%)	208 (46%)	781 (57%)	
URM	309 (56%)	82 (52%)	142 (43%)	375 (57%)	244 (54%)	586 (43%)	
Race / Ethnicity							<0.001
Asian	119 (22%)	33 (21%)	82 (25%)	135 (21%)	108 (24%)	333 (24%)	
Black	101 (18%)	26 (17%)	46 (14%)	115 (18%)	54 (12%)	131 (9.6%)	
Hispanic	178 (32%)	45 (29%)	69 (21%)	197 (30%)	162 (36%)	380 (28%)	
Native American	12 (2.2%)	4 (2.5%)	16 (4.8%)	19 (2.9%)	14 (3.1%)	32 (2.3%)	
Other	15 (2.7%)	6 (3.8%)	8 (2.4%)	38 (5.8%)	14 (3.1%)	32 (2.3%)	
PI	3 (0.5%)	1 (0.6%)	3 (0.9%)	6 (0.9%)	0 (0%)	11 (0.8%)	
White	120 (22%)	42 (27%)	109 (33%)	144 (22%)	100 (22%)	448 (33%)	
Sex							0.7
Male	181 (33%)	59 (38%)	102 (31%)	185 (28%)	135 (30%)	433 (32%)	
Female	354 (65%)	96 (61%)	224 (67%)	449 (69%)	298 (66%)	889 (65%)	
Other	13 (2.4%)	2 (1.3%)	7 (2.1%)	20 (3.1%)	19 (4.2%)	45 (3.3%)	

1 Statistics presented: n (%); Mean (SD).

2 Statistical tests performed: Fisher’s Exact Test for Count Data with simulated p-value (based on 2000 replicates); Kruskal-Wallis test.

* URE P refers to students who reported to have worked in a professor’s research project for 1 or more months and were part of a formal program.

** URE-nP students are those who responded to have worked in a professor’s research project for 1 or more months but were not part of a formal program.

*** No URE students are those who responded to have worked on a professor’s research project for zero months.

**TABLE 3:** Survey respondent characteristics based on type of URE participation and application to graduate or professional school.

Characteristic	Applied to graduate or professional schools			Did not apply to graduate or professional schools			*p*-values^2^
	URE-P*	URE-nP**	No URE***	URE-P	URE-nP	No URE	
	*N* = 548 (100%)^1^	*N* = 157 (100%)^1^	*N* = 333 (100%)^1^	*N* = 654 (100%)^1^	*N* = 452 (100%)^1^	*N* = 1,367 (100%)^1^	
Number of months research on professors project							<0.001
0 months	0 (0%)	0 (0%)	333 (100%)	0 (0%)	0 (0%)	1,367 (100%)	
1–3 months	54 (9.9%)	77 (49%)	0 (0%)	125 (19%)	264 (58%)	0 (0%)	
4–6 months	60 (11%)	39 (25%)	0 (0%)	102 (16%)	101 (22%)	0 (0%)	
7–12 months	61 (11%)	20 (13%)	0 (0%)	116 (18%)	42 (9.3%)	0 (0%)	
13–24 months	184 (34%)	12 (7.6%)	0 (0%)	170 (26%)	26 (5.8%)	0 (0%)	
25 + months	189 (34%)	9 (5.7%)	0 (0%)	141 (22%)	19 (4.2%)	0 (0%)	
Overall GPA							<0.001
A	308 (56%)	69 (44%)	163 (49%)	346 (53%)	174 (38%)	533 (39%)	
B	231 (42%)	69 (44%)	154 (46%)	261 (40%)	232 (51%)	725 (53%)	
C and D	9 (1.6%)	19 (12%)	16 (4.8%)	47 (7.2%)	46 (10%)	109 (8.0%)	
Biomedical major natural sciences	232 (42%)	62 (40%)	136 (41%)	281 (43%)	176 (39%)	513 (38%)	<0.001
Biomedical major social sciences	47 (8.6%)	5 (3.2%)	20 (6.0%)	53 (8.1%)	27 (6.0%)	72 (5.3%)	<0.001
Non-biomedical majors	269 (49%)	90 (57%)	177 (53%)	320 (49%)	249 (55%)	782 (57%)	<0.001
Science self-efficacy mean sum score (SD)	58 (9)	53 (11)	50 (11)	56 (9)	51 (11)	50 (11)	<0.001

1 Statistics presented: n (%); Mean (SD).

2 Statistical tests performed: Fisher’s Exact Test for Count Data with simulated p-value (based on 2000 replicates); Kruskal-Wallis test.

* URE P refers to students who reported to have worked in a professor’s research project for 1 or more months and were part of a formal program.

** URE-nP students are those who responded to have worked in a professor’s research project for 1 or more months but were not part of a formal program.

*** no URE students are those who responded to have worked on a professor’s research project for zero months.

**TABLE 4:** Model-based odds ratio estimates for research questions.

Research Question	URE-P OR (95% CI)	URE-nP OR (95% CI)	Non-URE OR (95% CI)
Research Question 1: Are graduate/professional school plans/application associated with URM vs non-URM status?	0.91 (0.72, 1.15)	1.175 (0.88, 1.50)	1.05 (0.78, 1.42)
Research Question 2: Are graduate/professional school plans/application associated with science self-efficacy?	1.12 (0.98, 1.27)	1.03 (0.91, 1.16)	0.99 (0.87, 1.13)
Research Question 3: Are graduate/professional school plans/applications associated with cumulative college GPA?			
a. A vs. B	non-URM: 1.15 (0.80, 1.66) URM: 1.07 (0.77, 1.50)	non-URM: 1.33 (0.89, 1.97) URM: 1.24 (0.85, 1.79)	non-URM: 1.50 (1.00, 2.24) URM: 1.40 (0.91, 2.15)
b. A vs. C, D	non-URM: 4.79 (1.85, 12.41) URM: 5.11 (2.08, 12.57)	non-URM: 0.80 (0.35, 1.80) URM: 0.85 (0.41, 1.78)	non-URM: 2.01 (0.76, 5.31) URM: 2.15 (0.87, 5.32)
c. B vs. C, D	non-URM: 4.15 (1.58, 10.89) URM: 4.76 (1.94, 11.68)	non-URM: 0.609 (0.26, 1.38) URM: 0.69 (0.33, 1.45)	non-URM: 1.34 (0.51, 3.57) URM: 1.54 (0.63, 3.78)

## Data Availability

The raw data supporting the conclusions of this article will be made available by the authors, without undue reservation.
