# Correction: Factors associated with applying to graduate/professional degrees for students engaged in undergraduate research experiences at minority serving institutions

**DOI:** 10.3389/feduc.2025.1650983

**Published:** 2025-08-11

**Authors:** Lourdes E. Echegoyen, Kala M. Mehta, Karsten Hueffer, Gabriela Chavira, Jacob D. Kagey, Thomas E. Keller, Kathleen M. Morgan, Stephen B. Aley, Chi-Ah Chun, Payam Sheikhattari, Amy Wagler

**Affiliations:** 1 Department of Chemistry & Biochemistry, Campus Office of Undergraduate Research Initiatives, The University of Texas at El Paso, El Paso, TX, United States; 2 Department of Epidemiology and Biostatistics, University of California San Francisco, San Francisco, CA, United States; 3 College of Natural Science and Mathematics, The University of Alaska Fairbanks, Fairbanks, AK, United States; 4 California State University, Northridge, CA, United States; 5 Department of Biology, University of Detroit Mercy, Detroit, MI, United States; 6 School of Social Work, Portland State University, Portland, OR, United States; 7 Department of Chemistry, Xavier University of Louisiana, New Orleans, LA, United States; 8 Department of Biological Sciences, The University of Texas at El Paso, El Paso, TX, United States; 9 Department of Psychology, California State University-Long Beach, Long Beach, CA, United States; 10 School of Community Health and Policy, Morgan State University, Baltimore, MD, United States; 11 Department of Public Health Sciences, The University of Texas at El Paso, El Paso, TX, United States

**Keywords:** undergraduate students, minority students, undergraduate research, applying to graduate/professional school, biomedical

In the published article, there was an error in the author list, and author Gabriela Chavira was erroneously excluded. The corrected author list appears below.

Lourdes E. Echegoyen, Kala M. Mehta, Karsten Hueffer, Gabriela Chavira, Jacob D. Kagey, Thomas E. Keller, Kathleen M. Morgan, Stephen B. Aley, Chi-Ah Chun, Payam Sheikhattari and Amy Wagler*

The affiliation of Gabriela Chavira is California State University, Northridge, United States

