# Supplementary material for: Factors associated with applying to graduate/professional degrees for students engaged in undergraduate research experiences at minority serving institutions
Source: Front Educ (Lausanne). Author manuscript; Available in PMC 2025 Oct 31. (PMC12574686; doi:10.3389/feduc.2025.1589105)
Supplement: Appendix A Supplementary Table 1 [file NIHMS2109671-supplement-Appendix_A_Supplementary_Table_1.docx]

**Appendix A: Items from CSS survey used in study**

To address our research questions, we selected the following items from the CSS:

- How many months since entering college (including summer) did you work on a professor’s research project? Possible responses are (in months): 0, 1-3, 4-6, 7-12, 13-24, and 25+.
- Mark your primary undergraduate major (multiple choices)
- Since entering college, have you participated in an undergraduate research program (Y/N)
- (Science Self-Efficacy Construct) How confident are you that you can

[Response categories: Absolutely, Very, Moderately, Somewhat, Not at All]

- - Use technical science skills (use of tools, instruments, and/or techniques)
  - Generate an answerable research question
  - Determine how to collect appropriate data
  - Explain the results of a study
  - Use scientific literature to guide research
  - Integrate results from multiple studies
  - Ask relevant questions
  - Identify what is known and not known about a problem
  - Understand scientific concepts
  - See connections between different areas of science and mathematics
- With respect to graduate or professional school, which best describes the current state of your educational plans? The top four answers were combined to indicate “Applied”. The bottom three were combined to indicate “did not apply”.
  - Accepted and will be attending in the fall
  - Accepted and deferred admission until a later date
  - Placed on waiting list, no acceptances
  - Still awaiting responses, no acceptances
  - Will be applying this coming fall
  - Not applying this fall, but might apply at a future date
  - No plans to apply to school now or in the future
- What is your current gender identity? (The last four options were bundled as “other” to simplify the analysis).
  - Man
  - Woman
  - Trans man
  - Trans woman
  - Gender queer/Gender non-conforming
  - Different identity (please state): [Free response]
- Are you (Select all that apply). Items 4-8 were bundled as “Asian” and items 10-12 were bundled as “Hispanic” to simplify the analysis.
  - White/Caucasian
  - African American/Black
  - American Indian/Alaska Native
  - East Asian (e.g., Chinese, Japanese, Korean, Taiwanese)
  - Filipino
  - Southeast Asian (e.g., Cambodian, Vietnamese, Hmong)
  - South Asian (e.g., Indian, Pakistani, Nepalese, Sri Lankan)
  - Other Asian
  - Native Hawaiian/Pacific Islander
  - Mexican American/Chicano
  - Puerto Rican
  - Other Latino
  - Other
